# High dose intravenous colistin methanesulfonate therapy is associated with high rates of nephrotoxicity; a prospective cohort study from Saudi Arabia

**DOI:** 10.1186/s12941-015-0062-8

**Published:** 2015-01-16

**Authors:** Ali S Omrani, Wafa A Alfahad, Mohamed M Shoukri, Abeer M Baadani, Sultan Aldalbahi, Ali A Almitwazi, Ali M Albarrak

**Affiliations:** Section of Infectious Diseases, Department of Medicine, King Faisal Specialist Hospital and Research Centre, PO Box 3354, MBC 46, Riyadh, 11211 Saudi Arabia; Department of Pharmacy, Prince Sultan Military Medical City, Riyadh, Saudi Arabia; Department of Biostatistics, King Faisal Specialist Hospital and Research Centre, Riyadh, Saudi Arabia; Department of Medicine, Prince Sultan Military Medical City, Riyadh, Saudi Arabia; Department of Pharmacy, Prince Sultan Cardiac Centre, Riyadh, Saudi Arabia; Division of Infectious Diseases, Department of Medicine, Prince Sultan Military Medical City, Riyadh, Saudi Arabia

**Keywords:** Colistin, Colistin methanesulfonate, CMS, Nephrotoxicity, Acute kidney injury, Saudi Arabia

## Abstract

**Background:**

Nephrotoxicity is an important adverse effect of colistin methanesulfonate (CMS) therapy. No data exist on rates and risk factors for colistin-related nephrotoxicity in Saudi Arabia (SA). We conducted a prospective cohort study to identify rates and risk factors for CMS nephrotoxicity in our patient population.

**Methods:**

We prospectively included adult patients who received ≥48 hours of intravenous CMS therapy. Pregnant patients and those on renal replacement were excluded. Patients received 9 million units (mU) loading dose followed by 3 mU 8 hourly. In renal impairment, CMS dosing was adjusted according to calculated creatinine clearance (CrCl). Nephrotoxicity was defined as per RIFLE criteria (Risk, Injury, Failure, Loss and End-stage renal disease). Statistical analysis was performed using SPSS version 20.0 (IBM, Armonk, New York, USA). The study was approved by the institution’s Research Ethics Committee.

**Results:**

A total of 67 patients were included in the study. Mean (±standard deviation) age was 57.5 (±24.0) years, Charlson Co-morbidity Score 2.88 (±2.39), CrCl 133.60 (±92.54) mL/min and serum albumin 28.65 (±4.45) g/L. Mean CMS dose was 0.11 (±0.04) mU/kg/day and mean total CMS dose received was 101.21 (±47.37) mU. Fifty-one (76.1%) patients developed RIFLE-defined nephrotoxicity. Mean total CMS dose and duration of therapy before onset of nephrotoxicity were 66.71 (±43.45) mU and 8.70 (±6.70) days, respectively. In bivariate analysis, patients with nephrotoxicity were significantly older (*P* 0.013) and had lower baseline serum albumin (*P* 0.008). Multivariate logistic regression identified serum albumin [odds ratio (OR) 0.72; 95% confidence interval (CI) 0.57–0.93; *P* 0.010] and intensive care admission (OR 16.38; 95% CI 1.37–195.55; *P* 0.027) as independent risk factors for CMS nephrotoxicity.

**Conclusions:**

High dose intravenous CMS therapy is associated with high rates of nephrotoxicity in SA. Independent risk factors for colistin nephrotoxicity were baseline hypoalbuminemia and intensive care admission.

## Background

Infections caused by carbapenem-resistant Gram-negative bacteria are becoming increasing more common, resulting in a parallel increase in the clinical use of intravenous colistin methanesulfonate (CMS), commonly known as colistin [1]. Data from pharmacokinetic studies suggest that standard CMS dosing regimens result in sub-optimal serum and target organ concentrations [2]. Moreover, without a high loading dose, it can take over 48 hours for colistin serum levels to reach steady state [3]. Many centres have changed their colistin dosing practice in an attempt to optimize antimicrobial treatment for patients with infections caused by carbapenem-resistant, Gram-negative bacteria [4].

Amongst the important potential adverse effects of CMS therapy are nephrotoxicity and neurotoxicity [5]. A number of risk factors for CMS-associated nephrotoxicity have been identified in different studies. However, it appears that CMS-related nephrotoxicity is reversible in the majority of cases and does not usually result in premature treatment discontinuation [5]. No data exist on the rates and risk factors for CMS-associated nephrotoxicity from Saudi Arabia or the Arabian Peninsula.

In May 2012, we introduced a CMS dosing protocol utilizing loading doses with higher maintenance doses for all patients requiring intravenous CMS therapy. The purpose of this study was to prospectively evaluate colistin-associated nephrotoxicity rates in our institution and to explore risk factors in our patient population.

## Methods

### Design

We conducted a prospective cohort study of adult patients starting targeted or empiric intravenous CMS therapy over the period from April 1 to September 30, 2013 in a large tertiary care centre in Riyadh, Saudi Arabia. The study was approved by Prince Sultan Military Medical City Research Ethics Committee and was granted a waiver for informed consent.

### Patients

We included all patients aged 18 years or more who received for ≥ 48 hours of intravenous CMS therapy. Exclusion criteria included current receipt of any form of renal replacement therapy, receipt of CMS therapy within the preceding 7 days, pregnancy, cystic fibrosis and extensive burns.

### Procedures

Baseline creatinine clearance (CrCl) was estimated using Cockroft-Gault equation [6]. Colistin was administered as Colomycin (Forest Laboratories, Bextley, United Kingdom), which is supplied in vials of 1 million units (mU) of CMS, equivalent to 80 mg CMS or 30 mg colistin base activity. Patients with normal renal function received 9 mU as a loading dose, followed 24 hours later by 3 mU every 8 hours. Adjustment in CMS dosing for patients with renal impairment, defined as estimated CrCl < 60 mL/min, was according to those recommended by Garonzik *et al.* [7]. We collected baseline demographic and clinical data including gender, age, body weight, site of infection and Charlson Co-morbidity Index [8]. Renal function and estimated CrCl were recorded on daily basis from start of CMS therapy up to 7 days after its discontinuation. Concomitant receipt of loop diuretics, aminoglycosides, vancomycin, amphotericin b preparations, non-steroidal anti-inflammatory agents or intravenous contrast was also recorded. CMS-related nephrotoxicity was defined according to RIFLE criteria [9] (Table 1).

**Table 1:** **RIFLE Criteria for acute kidney injury**

**Category**	**Criteria**
Risk **(R)**	Increased serum creatinine level by 1.5 times or GFR decrease by >25%
Injury **(I)**	Increased serum creatinine level by 2.0 times or GFR decrease by >50%
Failure **(F)**	Increased serum creatinine level by 3.0 times, GFR decrease by >75% or serum creatinine level ≥354 μmol/L
Loss **(L)**	Persistent acute renal failure or complete loss of function for >4 weeks
End-stage kidney disease **(E)**	End-stage kidney disease for >3 months

GFR = glomerular filtration rate.

### Outcomes

The primary end-point was the occurrence of RIFLE-defined nephrotoxicity whilst on intravenous CMS therapy. Secondary end-point was analysis of risk factors for CMS-associated nephrotoxicity.

### Statistical analysis

Bivariate analyses were performed using Fisher exact tests for categorical variables and the independent sample t test for continuous variables. Clinically important variables and those with *P* values of <0.2 in the bivariate analysis were included the multivariate logistic analysis. All *P* values were 2-sided and were considered significant if <0.05. Statistical analysis was performed using SPSS version 20.0 (IBM, Armonk, New York, USA).

## Results and discussion

**Figure 1:**
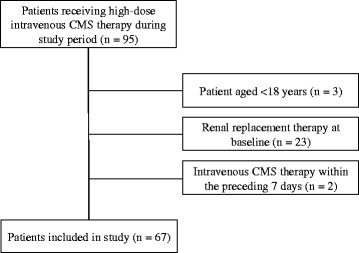
**Study population and patient selection.** CMS = Colistin methanesulfonate.

A total of 95 patients were started on high-dose intravenous CMS therapy during the study period, of which 67 met the inclusion criteria (Figure 1). Mean age (±standard deviation) was 57.48 (±24.01) years and 45 (67.2%) were males. Twenty-one patients (31.3%) were in an intensive care unit (ICU) and the average Charlson Co-morbidity Score was 2.88 (±2.39). At start of CMS therapy, mean estimated CrCl was 133.60 (±92.54) mL/min and serum albumin was 28.65 (±4.45) g/L. Sixteen patients (23.9%) had renal impairment (estimated CrCl < 60 mL/min) prior to starting CMS therapy and 29 (43.3%) had history of diabetes mellitus. Patients received an average intravenous CMS dose of 0.11 (±0.04) mU per kilogram per day, for an average duration of 13.76 (±6.77) days. The mean total CMS dose received was 101.21 (±47.37) mU. The underlying infections included hospital-acquired pneumonia (36, 53.7%), urinary tract infection (13, 19.4%), bacteraemia (7, 10.5%), skin and soft tissue infection (5, 7.5%) and other infections (6, 9.0%). Other baseline patient characteristics are summarized in Table 2.

**Table 2:** **Bivariate and multivariate logistic regression of risk factors for colistin methanesulfonate-associated nephrotoxicity**

	**Entire cohort (n = 67)**	**Nephrotoxicity group (n = 51)**	**No nephrotoxicity group (n = 16)**	**Bivariate analysis**	**Multivariate logistic regression**	
				**P value**	**OR (95% CI)**	**P value**
Age (years)^a^	57.48 (±24.01)	61.53 (±22.44)	44.56 (±24.99)	0.013	0.98 (0.96–1.04)	0.494
Body weight (kg)^a^	73.67 (±21.33)	72.78 (±18.90)	76.50 (±28.26)	0.547	-	-
Male gender^b^	45 (67.2%)	33(76.1%)	12 (75.0%)	0.55	-	-
Charlson Co-morbidity Score^a^	2.88 (±2.39)	2.86 (±2.14)	2.94 (±3.15)	0.914	-	-
Diabetes mellitus^b^	29 (43.2%)	25 (49.0%)	4 (25.0%)	0.15	0.52 (0.06–4.87)	0.568
Baseline CrCl (mL/min)^a^	133.60 (±92.54)	126.02 (±85.57)	157.94 (±111.52)	0.23	0.99 (0.98–4.87)	0.108
Baseline serum albumin (mg/L)^a^	28.65 (±4.45)	27.86 (±4.19)	31.19 (±4.48)	0.008	0.72 (0.57–0.93)	0.010
Intensive care unit care^b^	21 (31.3%)	13 (25.5%)	8 (50.0%)	0.12	16.38 (1.37–195.55)	0.027
Receipt of CMS Loading dose	52 (77.6%)	39 (76.5%)	13 (81.3%)	1.0	-	-
CMS Dose (mU/kg/day)^a^	0.11 (±0.04)	0.11 (±0.04)	0.10 (±0.04)	0.48	-	-
Cumulative CMS dose (mU)^a^	101.21 (±47.37)	106.47 (±45.91	84.44 (±49.56)	0.105	1.00 (1.00–1.00)	0.133
Duration of CMS therapy (days)^a^	13.76 (±6.77)	14.16 (±5.54)	12.50 (±9.86)	0.397	0.96 (0.83–1.11)	0.555
Number of concomitant nephrotoxic agents^a^	0.71 (±0.62)	0.76 (±0.65)	0.56 (±0.51)	0.26	2.37 (0.50–11.19)	0.277
Concomitant vancomycin therapy^b^	33 (49.3%)	24 (47.1%)	9 (56.3%)	0.576	-	-
Concomitant furosemide therapy^b^	11 (16.4%)	8 (15.7%)	3 (18.75%)	0.715	-	-
Concomitant aminoglycoside therapy^b^	3 (4.47%)	2 (3.92%)	1 (6.25%)	0.559	-	-
Concomitant NSAID^b^	2 (2.98%)	2 (3.92%)	0 (0.0%)	1.00	-	-
Concomitant radiological contrast^b^	1 (1.49%)	1 (1.96%)	0 (0.0%)	1.00	-	-
Concomitant amphotericin b therapy^b^	0 (0.0%)	0 (0.0%)	0 (0.0%)	1.00	-	-

CrCl = creatinine clearance; CMS = colistin methanesulfonate; OR = odds ratio; NSAID = Non-steroidal anti-inflammatory drugs.

^a^Mean (± standard deviation).

^b^Number (percentage).

A total of 51 (76.1%) patients developed RIFLE-defined CMS-related nephrotoxicity; 19 (37.3%) R; 20 (38.5%) I; and 12 (23.5%) F. Only 3 (5.9%) patients required renal replacement therapy. There were no treatment discontinuations secondary to nephrotoxicity.

The mean total CMS dose received before onset of nephrotoxicity was 66.71 (±43.45) mU and the mean duration of CMS therapy before onset of nephrotoxicity was 8.70 (±6.70) days. In bivariate analysis, patients who developed CMS-related nephrotoxicity were significantly older (*P* 0.013) and had lower baseline serum albumin (*P* 0.008). There were no other statistically significant differences between the two groups (Table 2). In multivariate logistic regression analysis, serum albumin [odds ratio (OR) 0.72; 95% confidence interval (CI) 0.57–0.93; P 0.010] and ICU status (OR 16.38; 95% CI 1.37–195.55; P 0.027) were the only independent risk factors for CMS-associated nephrotoxicity (Table 2). Renal function returned to baseline level within 7 days in 19 (37.3%) out of 51 patients who developed CMS-associated nephrotoxicity.

The realization that higher doses of CMS are required to optimize serum colistin concentration has been associated with concerns over an increased risk of nephrotoxicity. A few studies had reported the rates and risk factors for CMS-related nephrotoxicity, but ours is the first from the Arabian Peninsula region (Table 3) [10-20]. Whereas rates ranging between 17.8% and 53.5% were previously reported, just over 3 quarters (76.1%) of 67 adult patients included in our study developed acute kidney injury (AKI) whilst receiving high dose intravenous CMS therapy. Even though it has been suggested that using sensitive criteria such as RIFLE may result in higher rates of AKI, our rates are relatively high even when compared with studies that used the same definitions (Table 3). The reasons for such high rates of nephrotoxicity in our study are not clear. Baseline characteristics of our study population are comparable to those from previous studies and only 31.3% were critically ill. Furthermore, our dosing regimens are similar to some of those reported previously. It is noteworthy that despite the high rate of AKI in our study, only a small proportion of our patients (3, 5.9%) required renal replacement therapy and colistin therapy was not discontinued because of nephrotoxicity in any of the cases.

**Table 3:** **Summary of main studies in which rates and independent risk factors for colistin methanesulfonate sodium-associated nephrotoxicity were reported**

**Study and year published**	**Study design and population**	**Location**	**Was nephrotoxicity defined as per RIFLE criteria?**	**Rate of nephrotoxicity**	**Independent risk factors for CMS-associated nephrotoxicity**
Balkan et al., 2014 [10]	Retrospective cohort, 198 patients	Turkey	Yes	46.1%	Age > 60 years
Sorli et al., 2013 [11]	Prospective cohort; 102 patients	Spain	Yes	49.0%	Trough serum colistin level, Charlson Score and receipt of ≥ 2 concomitant nephrotoxic agents.
Dalfino et al., 2012 [12]	Prospective cohort; 28 ICU patients	Italy	No	17.8%	Receipt of radio-contrast.
Gauthier et al., 2012 [13]	Case–control; 370 patients with BMI > 25 kg/m^2^	United States	Yes	48%	BMI ≥ 31.5 kg/m^2^, diabetes mellitus, older age and length of hospital stay.
Doshi et al., 2011 [14]	Retrospective cohort; 49 ICU patients	United States	Yes	31%	Pre-existing chronic kidney disease, systemic hypertension, receipt of radio-contrast and receipt of ≥ 2 concomitant nephrotoxic agents.
Pogue et al., 2011 [15]	Retrospective cohort; 126 patients	United States	Yes	43%	Higher CMS dose, concomitant rifampicin therapy and receipt of ≥ 3 concomitant nephrotoxic agents.
Rattanaumpawan et al., 2011 [16]	Retrospective case control; 139 cases	Thailand	No	52.5%	Older age, longer duration of CMS therapy, higher CMS doses and concomitant vancomycin therapy.
Deryke et al., 2010 [17]	Retrospective cohort; 30 patients	United States	Yes	33%	Dosing based on actual body weight.
Kwon et al., 2010 [18]	Retrospective cohort; 71 patients	South Korea	Yes	53.5%	Male gender, concomitant use of a calcineurin inhibitor, hypoalbuminaemia and hyperbilirubinaemia.
Hartzell et al., 2009 [19]	Retrospective cohort; 66 patients	United States	Yes	45%	Cumulative CMS dose and longer duration of therapy.
Kim et al., 2009 [20]	Case–control; 47 cases	South Korea	Yes	31.9%	Hypoalbuminemia and concomitant use of non-steroidal anti-inflammatory drugs.

CMS = colistin methanesulfonate; RIFLE = Risk, Injury, Failure, Loss, End stage kidney disease; ICU = intensive care unit; BMI = body mass index.

Various risk factors for colistin-related nephrotoxicity were previously reported. However, only older age and baseline hypoalbuminemia were significantly different in our patients who developed AKI whilst on intravenous CMS therapy. There were no other statistically significant differences in rates of diabetes mellitus, baseline serum creatinine levels, ICU status, daily or cumulative CMS dose or duration of CMS therapy (Table 2). Moreover, our multivariate regression analysis identified lower baseline serum albumin levels and ICU status as the only independent risk factors for colistin-related AKI. Interestingly, serum albumin was included in 2 previous studies, both of which reported a significant association in multivariate analyses [18,20]. It had been suggested that AKI maybe related to higher free colistin concentrations as a result of lower protein binding [5,20]. From a clinical perspective, it would be of interest to investigate whether albumin replacement could reduce the risk of colistin-related nephrotoxicity in patients with lower albumin levels. On the other hand, ICU status was included in many previous studies but was not previously reported to have a significant independent association with colistin-related nephrotoxicity [13,15-17]. Contrary to our findings, some authors hypothesized that improved monitoring in ICU setting may contribute to lower rates of CMS-associated nephrotoxicity [5]. Overall, the literature has not been consistent on which variable are predictive of CMS nephrotoxicity (Table 3). This is probably due to the heterogeneity of the studies in terms of severity of illness of included patients, CMS dosing protocols and even definitions of AKI. For example, Hartzell et al. [19] included relatively younger patients with low co-morbidity scores, whereas others included only patients from ICU [14] or those with body mass indices of more than 25 kg/m^2^ [13].

Similar to many previous studies, our study is limited by the lack of a control group to better assess the effect of various variables. Moreover, it would have useful to include pharmacokinetic assessment to investigate the relationship between serum colistin levels and rates of nephrotoxicity. Finally, we noted that renal function recovered to baseline levels in over one third (37.3%) of patients with CMS-associated nephrotoxicity. Extending follow up to a period of 3 to 6 months could have helped understand the medium to long-term prognosis.

## Conclusions

High-dose intravenous CMS therapy is associated with high rates of nephrotoxicity in Saudi Arabia. The main independent risk factors in our patient population are low serum albumin at baseline and ICU status. Adequately controlled studies of appropriate size are required to better understand the factors associated with CMS-nephrotoxicity and identify potentially useful intervention to prevent its occurrence.

## Abbreviations

AKI: Acute kidney injury
BMI: Body mass index
CI: Confidence interval
CMS: Colistin methanesulfonate
CrCl: Creatinine clearance
GFR: Glomerular filtration rate
ICU: Intensive care unit
NSAID: Non-steroidal anti-inflammatory drugs
OR: Odds ratio
RIFLE: Risk, injury, failure, loss, end-stage kidney disease
mU: Million units
